# Survival from cancer of the rectum in England and Wales up to 2001

**DOI:** 10.1038/sj.bjc.6604579

**Published:** 2008-09-23

**Authors:** E Mitry, B Rachet, M J Quinn, N Cooper, M P Coleman

**Affiliations:** 1 Département d'Hépatogastroentérologie et Oncologie Digestive, Centre Hospitalo-Universitaire Ambroise-Paré, 9 avenue Charles de Gaulle, Boulogne, France; 2 Cancer Research UK Cancer Survival Group, Non-Communicable Disease Epidemiology Unit, Department of Epidemiology and Population Health, London School of Hygiene and Tropical Medicine, Keppel Street, London, UK; 3 Social and Health Analysis and Reporting Division, Office for National Statistics (Room FG/114), 1 Myddelton Street, London EC1R 1UW, UK

Rectal cancer is the fifth most frequent cancer in both sexes combined in England and Wales (Quinn *et al*, 2001). It is less common than colon cancer, but incidence trends during the 1990s were similar (Coleman *et al*, 1999). Improvements in survival have been slower in the United Kingdom than elsewhere in western Europe (Sant *et al*, 2001; Finn-Faivre *et al*, 2002; Martijn *et al*, 2003). There was a substantial deprivation gradient in survival for patients diagnosed with rectal cancer in England and Wales up to 1990, with patients in the most affluent group having 1-year and 5-year relative survival 5–7% higher than those in the most deprived group, even after adjustment for differences and trends in background mortality between these socioeconomic groups (Coleman *et al*, 1999).

Almost 156 000 patients were registered with cancer of the rectum (rectum (ICD-10 C20) and rectosigmoid junction (C19)) in England and Wales during the 14-year period 1986–1999, with a male–female sex ratio of 1.4 (range 1.3–1.6 between the eight English regions and Wales). Annual incidence rates in each deprivation category ranged from 16 to 20 per 100 000 during the 1990s, with no clear socioeconomic gradient. The proportion of rectal tumours recorded as adenocarcinoma increased from 65 to 75% by 1999, but a commensurate fall in the proportion of poorly specified carcinomas (from 25 to 15%) suggests that the quality of pathology data has improved, and the true proportion of adenocarcinoma was approximately 75% throughout the 1990s. Squamous carcinoma represented 4% of rectal tumours, unchanged since the late 1980s. Information on the stage of rectal cancer at diagnosis was not available in the national cancer registry before 1995, and stage-specific analyses for the period 1986–1999 were thus not possible. Approximately 7% of patients (regional range 2–12%) who were otherwise eligible for analysis were excluded because they were registered solely from a death certificate, so their duration of survival was unknown (zero recorded survival: date of diagnosis the same as the date of death). The proportion of cases with zero recorded survival was similar in all socioeconomic groups (5–6%, data not shown), however, so exclusions from analysis are unlikely to have had any impact on socioeconomic gradients in survival, or on changes in that gradient with time. The vital status of 2% of patients was unknown, and a further 3% were excluded because the rectal cancer was not their first primary malignancy. In all, 132 602 patients were included in the analyses (87.8% of those eligible).

## Survival trends

One-year, 5-year and 10-year relative survival rose substantially and significantly in both sexes between 1986–1990 and 1996–1999, by an average of 5–8% every 5 years, after adjustment for deprivation (Table 1). The increase in 5-year survival was particularly marked between the early and late 1990s, in both sexes.

**Figure 1:**
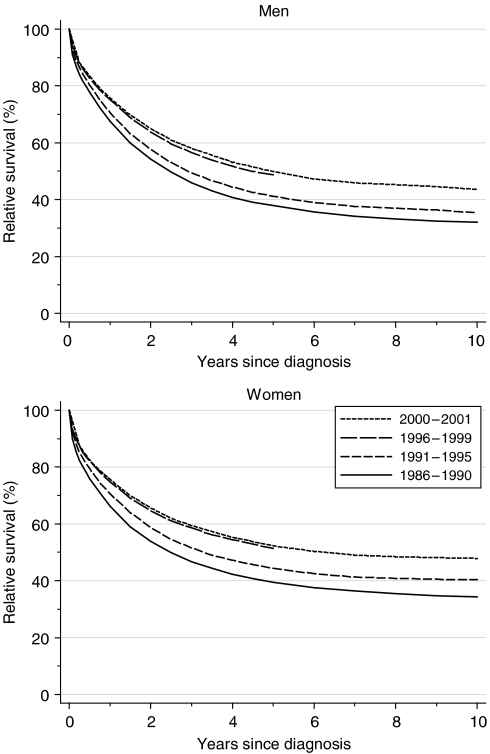
Relative survival (%) up to 10 years after diagnosis by sex and calendar period of diagnosis: England and Wales, adults (15–99 years) diagnosed during 1986–1999 and followed up to 2001. Survival estimated with cohort or complete approach (1986–1990, 1991–1995, 1996–1999) or hybrid approach (2000–2001) (see Rachet *et al*, 2008).

Short-term predictions of survival for patients diagnosed during 2000–2001, using hybrid analysis (Brenner and Rachet, 2004), do not suggest any substantial improvement in the near future (Table 1, Figure 1).

## Deprivation

**Figure 2:**
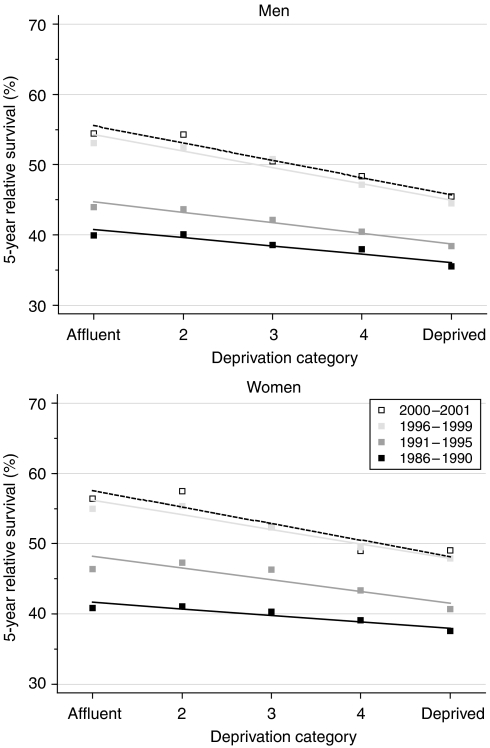
Trends in the deprivation gap in 5-year relative survival (%) by sex and calendar period of diagnosis: England and Wales, adults (15–99 years) diagnosed during 1986–1999 and followed up to 2001.

Survival increased for men and women in all deprivation groups to the end of the 1990s, but the increase was smaller in the more deprived groups in both sexes, and the deprivation gap in survival widened as a result (Table 2, Figure 2). Trends in the deprivation gap over time were similar in both sexes. For patients diagnosed during 1986–1990 and 1996–1999, respectively, the deprivation gap in 5-year relative survival widened from −5% to −9% in men (weighted average change −2.4% every 5 years) and from −4% to −8% in women (−2.5% every 5 years), even after adjustment for increasing differences in background mortality between the deprivation groups. Short-term prediction with hybrid analysis suggests that the widening of the deprivation gap is likely to continue in the near future (Table 2).

## Comments

There has been a dramatic improvement in rectal cancer survival over the last 15 years, particularly during the 1990s. Substantial advances in rectal cancer management have occurred during the last two decades. Earlier diagnosis has allowed more effective treatment (increase in resection rates, especially sphincter-saving procedures) and a substantial reduction in operative mortality (Finn-Faivre *et al*, 2002; Mitry *et al*, 2002). Major new developments have also taken place in treatment, including total mesorectal excision and adjuvant radiotherapy (Kapiteijn *et al*, 2002; Martijn *et al*, 2003). Together, these are likely to explain the increasing overall trends in survival. Improvements in survival in England and Wales have been more marked than for colon cancer, but, as for colon cancer, improvements have mainly been confined to patients in the most affluent groups. The deprivation gradient in rectal cancer survival (8–9% at 5 years) is greater than for colon cancer, and the significant increase seen during the 1990s suggests that the most deprived patients have not benefited equally from the optimal treatment currently available.

## Figures and Tables

**Table 1:** Trends in relative survival (%) by sex, time since diagnosis and calendar period of diagnosis: England and Wales, adults (15–99 years) diagnosed during 1986–1999 and followed up to 2001

		**Calendar period of diagnosis^a^**						**Average change (%)**		**Prediction^c^ for patients**	
		**1986–1990**		**1991–1995**		**1996–1999**		**every 5 years^b^**		**diagnosed during 2000–2001**	
**Time since diagnosis**		**Survival (%)**	**95% CI**	**Survival (%)**	**95% CI**	**Survival (%)**	**95% CI**	**Survival (%)**	**95% CI**	**Survival (%)**	**95% CI**
1 year	Men	**67.5**	(66.9, 68.2)	**70.6**	(70.1, 71.2)	**75.2**	(74.6, 75.7)	**5.2** ^**^	(4.0, 6.4)	**75.8**	(75.0, 76.6)
	Women	**66.2**	(65.5, 66.9)	**70.6**	(69.9, 71.3)	**74.7**	(74.0, 75.4)	**5.5** ^**^	(4.1, 6.9)	**75.6**	(74.6, 76.6)
5 years	Men	**37.8**	(37.1, 38.6)	**41.1**	(40.5, 41.8)	**48.7**	(47.8, 49.6)	**7.4** ^**^	(5.8, 8.9)	**49.8**	(48.8, 50.9)
	Women	**39.4**	(38.6, 40.2)	**44.3**	(43.5, 45.1)	**51.3**	(50.3, 52.4)	**8.1** ^**^	(6.3, 10.0)	**52.3**	(51.0, 53.5)
10 years	Men	**32.1**	(31.3, 32.9)	**35.4**	(34.5, 36.3)			**7.4** ^**^	(4.3, 10.5)	**43.6**	(42.3, 44.9)
	Women	**34.2**	(33.4, 35.1)	**40.3**	(39.4, 41.3)			**7.4** ^**^	(4.0, 10.8)	**47.9**	(46.4, 49.3)

CI=confidence interval.

a Survival estimated with cohort or complete approach (see Rachet *et al*, 2008).

b Mean absolute change (%) in survival every 5 years, adjusted for deprivation (see Rachet *et al*, 2008).

c Survival estimated with hybrid approach (see Rachet *et al*, 2008).

^**^P<0.01.

**Table 2:** Trends in the deprivation gap in relative survival (%) by sex, time since diagnosis and calendar period of diagnosis: England and Wales, adults (15–99 years) diagnosed during 1986–1999 and followed up to 2001

		**Calendar period of diagnosis^a^**						**Average change (%)**		**Prediction^c^ for patients**	
		**1986–1990**		**1991–1995**		**1996–1999**		**every** **5 years^b^**		**diagnosed during 2000–2001**	
**Time since diagnosis**		**Deprivation gap (%)**	**95% CI**	**Deprivation gap (%)**	**95% CI**	**Deprivation gap (%)**	**95% CI**	**Deprivation gap (%)**	**95% CI**	**Deprivation gap (%)**	**95% CI**
1 year	Men	−5.4^**^	(−7.3, −3.6)	−4.8^**^	(−6.4, −3.1)	−8.1^**^	(−9.7, −6.4)	−1.4^*^	(−2.7, −0.1)	−9.7^*^	(−12.0, −7.4)
	Women	−4.6^**^	(−6.7, −2.5)	−4.4^**^	(−6.4, −2.5)	−6.9^**^	(−9.0, −4.9)	−**1.2**	(−2.8, 0.3)	−5.7^*^	(−8.5, −2.9)
5 years	Men	−4.7^**^	(−6.8, −2.6)	−6.0^**^	(−8.0, −4.0)	−9.4^**^	(−12.0, −6.8)	−2.4^**^	(−4.1, −0.6)	−9.8^*^	(−13.0, −6.7)
	Women	−3.7^**^	(−6.1, −1.3)	−6.7^**^	(−9.0, −4.3)	−8.3^**^	(−11.4, −5.2)	−2.5^*^	(−4.5, −0.5)	−9.4^*^	(−13.1, −5.7)
10 years	Men	−3.6^**^	(−6.0, −1.3)	−8.7^**^	(−11.3, −6.2)			−5.1^**^	(−8.5, −1.7)	−11.2^*^	(−14.9, −7.5)
	Women	−3.9^**^	(−6.5, −1.4)	−5.8^**^	(−8.6, −3.0)			−**1.9**	(−5.7, 1.9)	−7.9^*^	(−12.1, −3.6)

CI=confidence interval.

a Survival estimated with cohort or complete approach (see Rachet *et al*, 2008).

b Mean absolute change (%) in the deprivation gap in survival every 5 years, adjusted for the underlying trend in survival (see Rachet *et al*, 2008).

c Survival estimated with hybrid approach (see Rachet *et al*, 2008).

^*^P<0.05; ^**^P<0.01.
